# The Role of Codon Usage, tRNA Availability, and Cell Proliferation in EBV Latency and (Re)Activation

**DOI:** 10.1055/s-0042-1751301

**Published:** 2022-09-15

**Authors:** Darja Kanduc

**Affiliations:** 1 Department of Biosciences, Biotechnologies and Biopharmaceutics, University of Bari, Bari, Italy

**Keywords:** EBNA1 translation, GAR, codon usage, tRNA availability, cell proliferation, adjuvants

## Abstract

Epstein–Barr nuclear antigen 1 (EBNA1) protein synthesis is inhibited during Epstein–Barr virus (EBV) latency and is resumed in EBV (re)activation. In analyzing the molecular mechanisms underpinning the translation of *EBNA1* in the human host, this article deals with two orders of data. First, it shows that the heavily biased codon usage of the *EBNA1* open reading frame cannot be translated due to its noncompliance with the human codon usage pattern and the corresponding tRNA pool. The *EBNA1* codon bias resides in the sequence composed exclusively of glycine and alanine, i.e., the Gly-Ala repeat (GAR). Removal of the nucleotide sequence coding for GAR results in an *EBNA1* codon usage pattern with a lower codon bias, thus conferring translatability to EBNA1. Second, the data bring cell proliferation to the fore as a conditio sine qua non for qualitatively and quantitatively modifying the host's tRNA pool as required by the translational needs of EBNA1, thus enabling viral reactivation. Taken together, the present work provides a biochemical mechanism for the pathogen's shift from latency to (re)activation and confirms the role of human codon usage as a first-line tool of innate immunity in inhibiting pathogens' expression. Immunologically, this study cautions against using codon optimization and proliferation-inducing substances such as glucocorticoids and adjuvants, which can (re)activate the otherwise quiescent, asymptomatic, and innocuous EBV infection. Lastly, the data pose the question whether the causal pathogenic role attributed to EBV should instead be ascribed to the carcinogenesis-associated cellular proliferation.

## Introduction

Epstein–Barr virus (EBV) is associated with infectious mononucleosis, multiple sclerosis, and numerous types of cancer. Epidemiologically, EBV infects 95% of individuals worldwide, and currently, the extent of the pathological burden is highest, with EBV causally linked to 200,000 cases of cancer each year and approximately 1.8% of total cancer-related deaths annually. 1 2 3 However, these numbers—although already impressive in themselves—define only a minimal part of the risks that might associate with EBV infection. Indeed, EBV is ubiquitous in the human population, mainly as an asymptomatic, harmless, latent infection, with only occasional reactivation, which is the harbinger of the EBV-related diseases. This means that if EBV reactivation occurred extensively in the human population, the number and the intensity of EBV-related diseases would increase substantially. In light of such a perspective, understanding the mechanism(s) that dictate and regulate EBV latency/reactivation is a fundamental research priority to prevent a possible wave of EBV-related pathologies.

Numerous factors appear to contribute to determine the EBV latency/(re)activation. Some examples include the following:

- Epigenetic machinery such as DNA methylation, host histone chaperones, m6A RNA modification, and nonsense-mediated decay. 4
- Psychological and cellular stressors resulting in EBV reactivation. 5
- Reactive oxygen species production in cells. 5
- Immune escape strategies. 6

At the molecular level, research mainly focused on Epstein–Barr nuclear antigen 1 (EBNA1), the expression of which characterizes all the four latent forms of EBV infection, i.e., I, IIa, IIb, and III, 7 8 9 10 thus suggesting crucial roles of this protein in EBV latency. Intriguingly, *EBNA1* is also essential for EBV reactivation because the viral protein initiates the EBV replication by binding to specific sites in *ori* P, the plasmid origin. 11 12 Additionally, *EBNA1* is highly antigenic, and so a dominant line of thought has been that EBV escapes from immune surveillance by limiting *EBNA1* protein production to minimal levels that cannot evoke immune responses. 13 14 15 16

Biochemically, such inhibition of *EBNA1* protein synthesis resides in a repetitive sequence composed of Gly and Ala residues, i.e., the *EBNA1* Gly-Ala repeat (GAR). In fact, in 2003, Yin et al 15 clearly proved that deletion of the repeat leads to a high level of *EBNA1* protein in human lung carcinoma cells.

However, despite such intense research and notwithstanding numerous hypotheses, 16 17 18 19 20 21 22 23 three fundamental questions remain unanswered. (1) Why and how *EBNA1* protein synthesis is restricted during latency? (2) Why and how *EBNA1* protein synthesis is resumed during EBV reactivation? (3) What is the underlying molecular mechanism of the GAR inhibitory effect?

Here, these issues are analyzed starting from the fact that a high level of molecular mimicry exists between human and pathogen proteins, 24 25 26 including EBV proteins, 27 28 29 with a consequent potential cross-reactivity and autoimmunity.

According to this molecular mimicry–oriented paradigm, the research rationale of the present work is based first on the consideration that, due to the numerous peptide commonalities, inhibition of pathogen protein synthesis during latency may represent a host device to avoid potential destructive autoimmune cross-reactions. 30 In the case in point, constraining the expression of *EBNA1* would prevent the host immune response against the *EBNA1* protein as well as cross-reactive reactions with the host proteins that share peptide sequences with the virus, 27 28 29 thus possibly explaining why *EBNA1* protein synthesis is repressed.

Second, this study searches for possible mechanisms underlying the inhibition of *EBNA1* protein synthesis on the basis of previous data 30 31 32 33 that suggested that the synthesis of a protein does not occur if the codon usage of the open reading frame (ORF) coding for the protein does not comply with the codon usage of the host. In this regard, data were obtained for the ORFs coding for (re)activation-related proteins from Herpes simplex virus type 1, 30 *Toxoplasma gondii* , 30 *Plasmodium falciparum* , 30 *Cryptococcus neoformans* , 30 *Cytomegalovirus* (CMV), 31 32 and severe acute respiratory syndrome coronavirus 2. 33

Therefore, as a logical extension of such data, the issues of why and how *EBNA1* protein synthesis is restricted/resumed during latency and (re)activation, and the role of *EBNA1* GAR have been analyzed through the lens of the compliance to the human codon usage.

## Materials and Methods

Codon usage analyses were conducted on the ORF of the EBNA1 gene from EBV B95-8 ( www.ncbi.nlm.nih.gov/nuccore/V01555.2 ), a type 1 strain, which is prevalent worldwide. 34 *EBNA1* ORF without the GAR sequence was obtained by deleting the genome nucleotide sequence position 108217–108924.

ORF of the human paired box PAX5 gene (NCBI accession: NM_016734.3, https://www.ncbi.nlm.nih.gov/nuccore/NM_016734.3 ) was used as a control as PAX5 protein is involved in EBNA1-driven transcription. 35

Codon usage analyses were performed using GeneInfinity program ( http://www.geneinfinity.org ). Codon usage of the *Homo sapiens* ORFeome (40,662,582 codons) was obtained from the international DNA sequence database ( http://www.kazusa.or.jp/codon/ ). ^36^ Codon usage for each codon is given as frequency per thousand. Amino acids (AA) are given in one- or three-letter code.

## Results and Discussion

### Human versus *EBNA1* Codon Usage

**Fig. 1:**
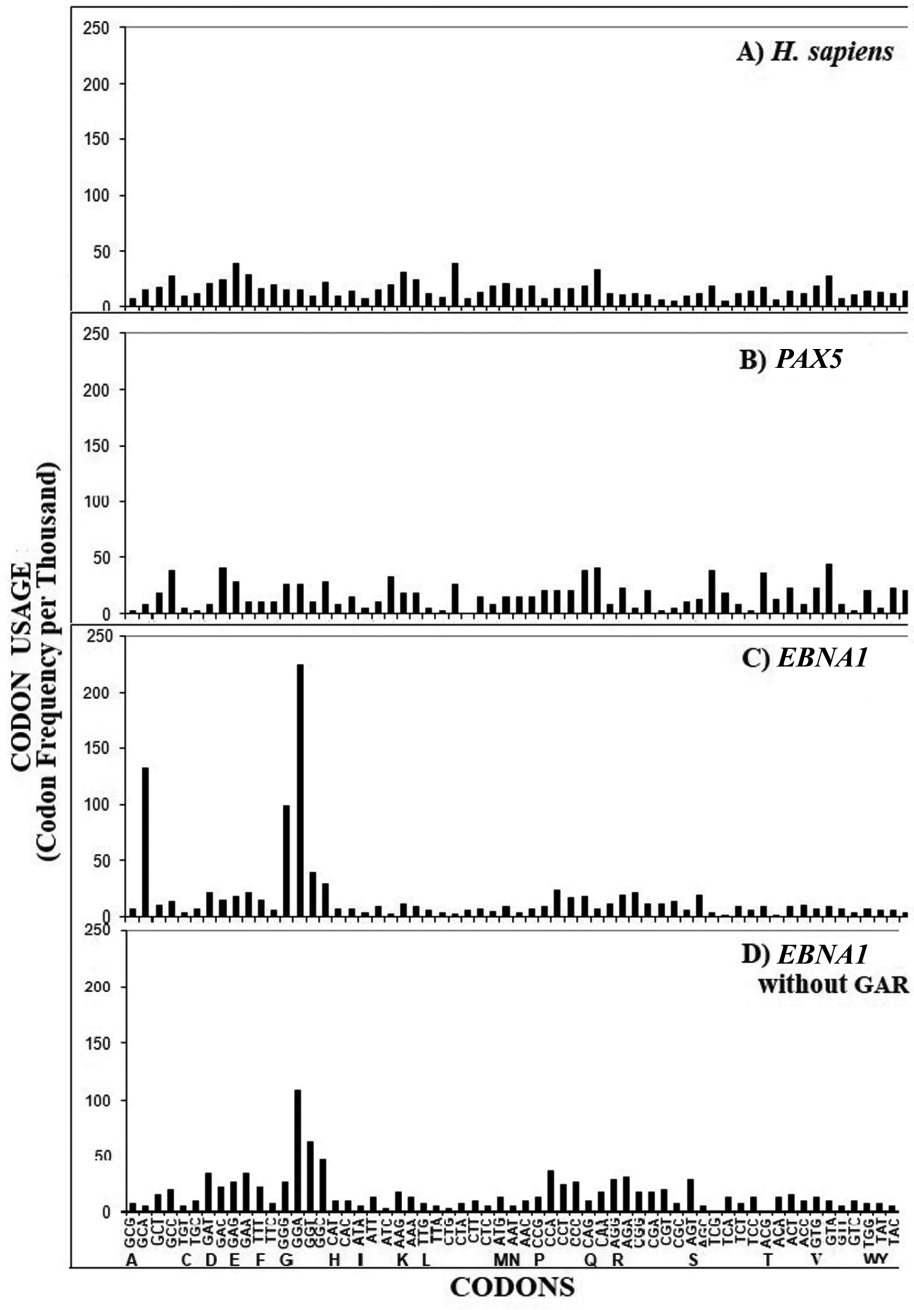
Codon usage of ( **A** ) human ORFeome, ( **B** ) human *PAX5* ORF, ( **C** ) *EBNA1* ORF, and ( **D** ) *EBNA1* ORF without the nucleotide sequence coding for GAR.

Fig. 1A–D shows the frequency per thousand of the 61 codons in the human ORFeome and in the ORFs coding for the human control PAX5, EBNA1, and EBNA lacking the GAR nucleotide sequence, respectively. Numerically, data illustrated in Fig. 1 are tabulated in Supplementary Table S1 .

Three main points stand out from Fig. 1 :

- The codon usage of the human *PAX5* ORF ( Fig. 1B ) complies with the codon usage of the human ORFeome ( Fig. 1A ), thus following the fundamental principle according to which each gene in a genome tends to conform to its species' codon usage pattern. 37 38
- In contrast, the codon usage of the ORF coding for *EBNA1* is markedly different from that of the human ORFeome and is characterized by a heavily biased codon usage pattern ( Fig. 1C vs Fig. 1A ). In practice, coding of Gly and Ala in the long nucleotide sequence corresponding to *EBNA1* GAR (i.e., position 108217–108924 in the EBV genome) is mostly delegated to three codons, i.e., GGG (Gly), GGA (Gly), and GCA (Ala), in front of the possible eight synonymous codons—four for each—that code for Gly and Ala.
- Deletion of the *EBNA1* nucleotide sequence coding for GAR decreases the bias degree of *EBNA1* codon usage pattern ( Fig. 1D vs Fig. 1C ).

Furthermore, it is of note that the codon usage of *EBNA2* , EBNA3, EBNA4, EBNA5, and EBNA6 showed, although to a lesser extent than that present in the *EBNA1* ORF, a certain degree of codon bias compared with the human codon usage (see Supplementary Fig. S1 ).

### Codon Usage Bias and tRNA Availability: Translational Regulation of EBNA1

The data displayed in Fig. 1 provide a key for understanding the biochemical mechanism by which *EBNA1* and its repeat allow the long-term persistence of the EBV genome. Indeed, according to a basic notion known since the 1980s, 37 38 the degree of biased codon usage is proportional to the production levels of individual genes, with highly expressed genes using only a small subset of codons, i.e., exhibiting greater codon bias compared with poorly expressed genes. Therefore, theoretically, the highly biased *EBNA1* codon usage ( Fig. 1C ) is apt to ensure an abundant production of *EBNA1* protein. On the other hand, such *EBNA1* codon optimization has no effect on the translational efficiency in the human host. Actually, Ikemura 39 and Ikemura and Ozeki 40 demonstrated that genes characterized by biased codon usage can be efficiently translated only in the presence of a specularly biased tRNA population. That is, codon usage and tRNA availability are functionally coadapted to each other in determining gene translation to protein.

In the case at issue, the profile of the tRNA pool quantitatively and qualitatively matches the pattern of the human codon usage, but not the highly biased codon usage of the viral EBNA1. In the human host, the foreign *EBNA1* ORF has no chance to be translated by being unavailable the biased tRNA profile corresponding to the biased *EBNA1* codon usage.

On this subject, it is worth recalling that viral ORFs characterized by suboptimal codon usage, i.e., populated by codons rarely used in the human codon usage, are likewise expected to remain untranslated in the host. This is the case of human CMV latency, which is characterized by restriction of viral protein synthesis. 32 In fact, in analyzing the molecular factors that hinder CMV expression in the human host, it was previously showed that the CMV genes frequently use six codons that are rarely used in the human host and that, in some instances, the rare host codons are clustered in viral nucleotide sequences coding for single AA repeats, thus posing extra translational constraints to CMV expression. 32

### Modifying the tRNA Pool: Cell Proliferation as a Primary Factor

In light of the above, the fundamental question of what is the mechanism that leads to resume *EBNA1* protein synthesis and, consequently, determines EBV (re)activation becomes the following one: what is the mechanism able to change the host tRNA pool according to the *EBNA1* translational needs? To this author's knowledge, 41 42 43 44 45 46 47 a primary process capable of removing the inhibition of *EBNA1* translation is represented by cell proliferation.

Indeed, the tRNA profile sharply changes in the human host during cell proliferation induced, for example, by partial hepatectomy or cancer 41 42 43 44 45 46 47 so that different tRNA populations characterize quiescence and proliferation, with quantitative increases in minor tRNAs scarcely expressed during quiescence and, vice versa, decreases in tRNAs abundantly expressed during the quiescent phase.

**Fig. 2:**
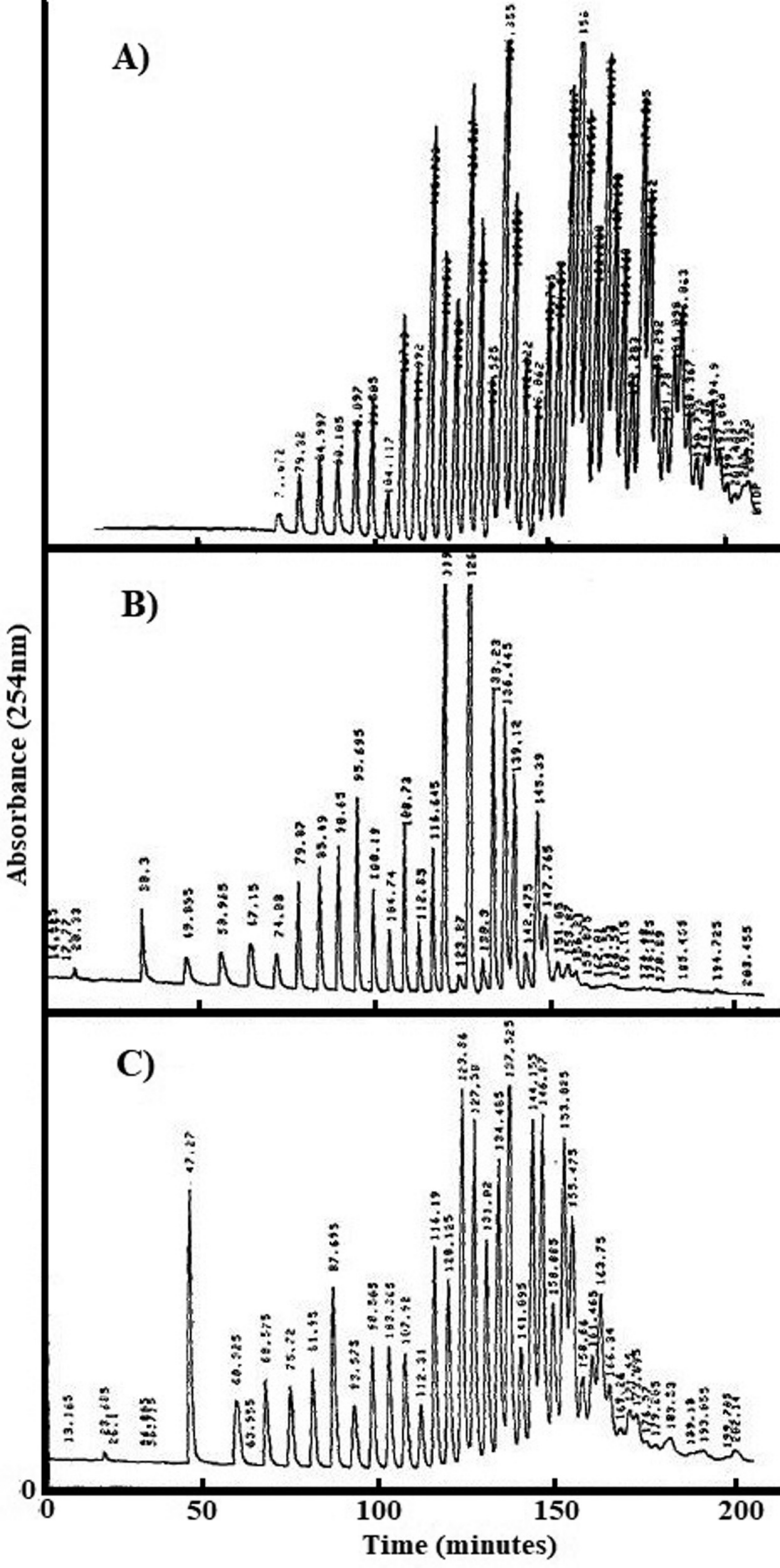
HPLC profile of tRNA pools in gastric and colorectal tumors. tRNAs from: ( **A** ) control colon tissue from obese resected patient and ( **B** ) gastric cancer tissue and ( **C** ) colorectal cancer tissue from cancer patients. (Reproduced with permission from Kanduc et al. 45 )

An example of the tight relationship between proliferation and changes of the tRNA profile is illustrated in Fig. 2 where a visual representation is given of the different tRNA patterns that characterize human gastric and colorectal carcinomas ( Fig. 2B,C ) compared with noncancer nonproliferating control tissue ( Fig. 2A ). 45 Of note, data from Fig. 2 assume a relevant significance regarding the *EBNA1* translation given that tRNAs ^Ala^ and tRNAs ^Gly^ represent the main tRNAs involved in the qualitative and quantitative changes of the tRNA profile in human gastric and colorectal tumors, as detailed by Kanduc et al. 45

Then, Fig. 2 might mechanistically explain the *EBNA1* protein synthesis obtained by Yin et al 15 using human lung carcinoma cells and deleting *EBNA1* GAR. That is, *EBNA1* protein synthesis became possible since human lung carcinoma cells supplied the oncoproliferative cellular context for inducing tRNAs not available in the human quiescent host, while deletion of the nucleotide sequence coding for GAR modified the highly biased *EBNA1* codon usage to a less biased and potentially more translatable pattern.

Hence, Fig. 2 also poses the issue of the causal link between EBV infection and cancer. That is, since EBV and gastric cancer represent the most common form of EBV-associated neoplasm 48 and EBV is significantly associated with colorectal cancer, 49 from a logical point of view it is justified to hypothesize that the causal pathogenic role—currently attributed to EBV—should be ascribed to the carcinogenesis-associated proliferation. Indeed, by inducing tRNA patterns able to favor EBV translation, oncoproliferation might cause, in the following order: EBV protein expression, host's anti-EBV immune responses, cross-reactivity with host's proteins sharing peptide sequences with EBV, 27 28 29 and, as a logical final consequence, numerous diseases from lymphomas to lupus and multiple sclerosis. 28

The fundamental role of cell proliferation in EBV (re)activation is also supported by the facts that: (1) EBV latency and lytic gene expression may be modulated by epigenetic mechanisms 4 such as DNA hypomethylation, a ubiquitous feature of cellular (onco)proliferation, 50 51 52 and (2) cellular and viral DNA hypomethylation are known to induce EBV lytic cycle, 53 and indeed azacytidine, which is a DNA methyltransferase inhibitor, rapidly activates the EBV lytic cycle. 54

## Conclusions

This study analyzes the factors that may underlie the inhibition of *EBNA1* protein synthesis during latency and highlights the fundamental role of cell proliferation for adapting qualitatively and quantitatively the human tRNA pool to the translational needs of EBNA1.

Indeed, the requirement of functional coadaptation between the pattern of a gene codon usage and the abundance of tRNA species is so tight 39 40 that, from an evolutionary point of view, it substantiates the concept that the codon usage pattern of the various (micro)organisms, tissues, and cells has been selected to be specifically adapted to the tRNA profiles of the (micro)organisms, tissues, and cells, instead of the tRNA pool having been adapted as a function of the codon usage profiles. 55 56

Clinically, the data exposed here might help understand the issue of EBV reactivation during pregnancy and in fetuses and newborns, 57 58 59 i.e., in rapidly proliferating organisms, as well as in subjects treated with immunosuppressive drugs, for example, after organ transplant. De facto, it is well known that glucocorticoids promote cell proliferation 60 61 62 63 64 65 66 67 68 69 70 71 and, consequently, can induce tRNA changes favoring EBV (re)activation.

Immunologically, it is noteworthy that the mechanism of the adjuvants in active immunization consists in stimulating powerful B- and T-cell proliferation. 72 73 74 This means that adjuvant-induced proliferation might lead to changes in the cellular tRNA pools, thus opening the door to reactivation of latent and inherently harmless infections with consequent pathologic sequelae such as autoimmune cross-reactivity. This risk appears even more menacing considering that latent EBV infection is present in 95% of the human population. Then, according to the data discussed, prophylactic/therapeutic campaigns of anti-EBV vaccination would be possible only if based on the concept of peptide uniqueness, i.e., on peptides unique to the viral plural: proteins and absent in the human host. 75
