# Supplementary material for: The Role of Codon Usage, tRNA Availability, and Cell Proliferation in EBV Latency and (Re)Activation
Source: Glob Med Genet. 2022 Sep 15;9(3):219–25. doi: 10.1055/s-0042-1751301 (PMC9477563; doi:10.1055/s-0042-1751301)
Supplement: Supplementary file 1 — Supplementary Material [file 10-1055-s-0042-1751301-s2200021.pdf]

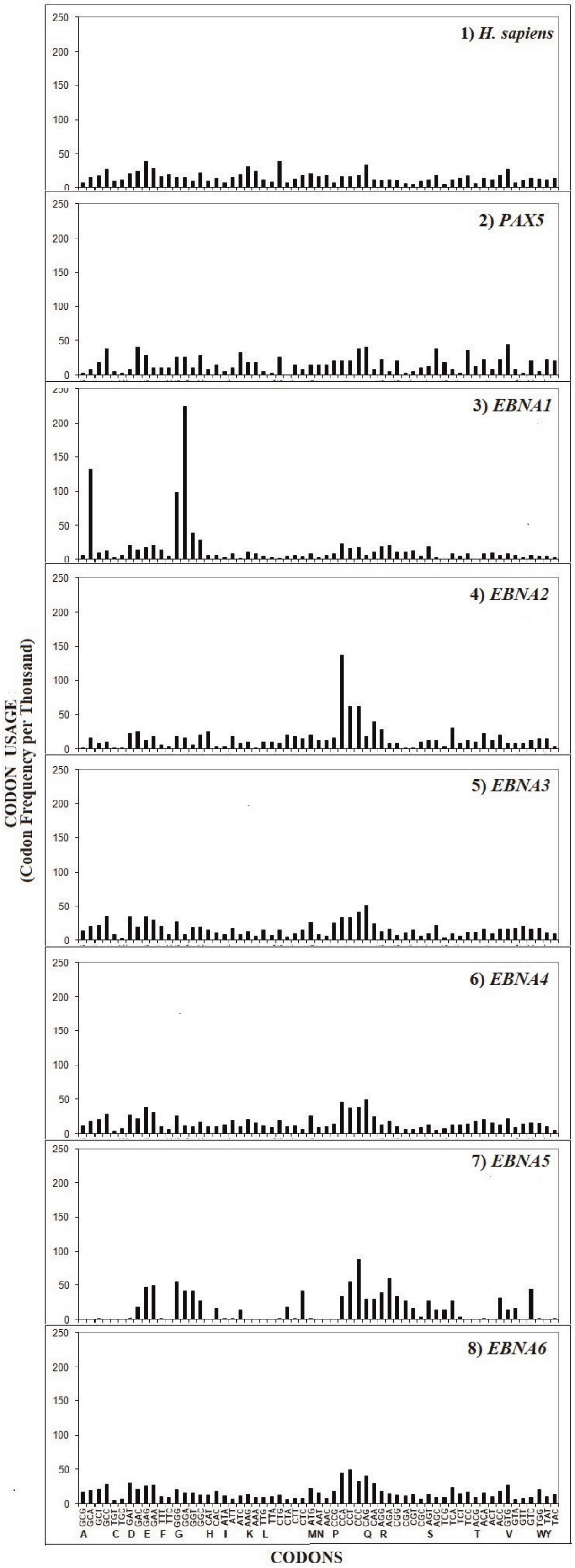

Supplementary Fig. S1 Codon usage of (1) human ORFeome, (2) human *PAX5* ORF, and (3 to 8) *EBNA1* ORF to *EBNA6* ORF.

 © 2022. The Author(s).

Supplementary Table S1 Codon usage of the human ORFeome and of ORFs coding for *PAX5*, *EBNA1*, and *EBNA1* without GAR[a]

aa	Codon	*H. sapiens*	*PAX5*	*EBNA1*	*EBNA1*[b]
Ala	GCG	7.4	2.5	6.2	7.4
Ala	GCA	15.8	7.6	132.4	4.9
Ala	GCT	18.4	17.9	9.3	14.8
Ala	GCC	27.7	38.3	12.5	19.7
Cys	TGT	10.6	5.1	3.1	4.9
Cys	TGC	12.6	2.5	6.2	9.8
Asp	GAT	21.8	7.6	21.8	34.5
Asp	GAC	25.1	40.8	14.0	22.2
Glu	GAG	39.6	28.1	17.1	27.1
Glu	GAA	29.0	10.2	21.8	34.5
Phe	TTT	17.6	10.2	14.0	22.2
Phe	TTC	20.2	10.2	4.7	7.4
Gly	GGG	16.5	25.5	98.1	27.1
Gly	GGA	16.5	25.5	224.3	108.4
Gly	GGT	10.7	10.2	38.9	61.6
Gly	GGC	22.2	28.1	29.6	46.8
His	CT	10.9	7.6	6.2	9.8
His	CAC	15.1	15.3	6.2	9.8
Ile	ATA	7.5	5.1	3.1	4.9
Ile	ATT	16.0	10.2	7.8	12.3
Ile	ATC	20.8	33.2	1.6	2.5
Lys	AAG	31.9	17.9	10.9	17.2
Lys	AAA	24.4	17.9	7.8	12.3
Leu	TTG	12.9	5.1	4.7	7.4
Leu	TTA	7.7	2.5	3.1	4.9
Leu	CTG	39.6	25.5	1.6	2.5
Leu	CTA	7.1	0.0	4.7	7.39
Leu	CTT	13.2	15.3	6.3	9.8
Leu	CTC	19.6	7.6	3.2	4.9
Met	ATG	22.0	15.3	7.8	12.3
Asn	AAT	17.0	15.3	3.1	4.9
Asn	AAC	19.1	15.3	6.2	9.8
Pro	CCG	6.9	20.4	7.8	12.3
Pro	CCA	16.9	20.4	23.4	36.9
Pro	CCT	17.5	20.4	15.6	24.6
Pro	CCC	19.8	38.3	17.1	27.1
Gln	CAG	34.2	40.8	6.2	9.8
Gln	CAA	12.3	7.6	10.9	17.2
Arg	AGG	12.0	23.0	18.7	29.5
Arg	AGA	12.2	5.1	20.2	32.0
Arg	CGG	11.4	20.4	10.9	17.2
Arg	CGA	6.2	2.5	10.9	17.2
Arg	CGT	4.5	5.1	12.5	19.7
Arg	CGC	10.4	10.2	4.7	7.4

(*Continued*)

Supplementary Table S1 (*Continued*)

aa	Codon	*H. sapiens*	*PAX5*	*EBNA1*	*EBNA1*[b]
Ser	AGT	12.1	12.7	18.7	29.6
Ser	AGC	19.5	38.3	3.1	4.9
Ser	TCG	4.4	17.9	0.0	0.0
Ser	TCA	12.2	7.6	7.8	12.3
Ser	TCT	15.2	2.5	4.7	7.4
Ser	TCC	18.0	35.7	7.8	12.3
Thr	ACG	6.0	13.0	0.0	0.0
Thr	ACA	15.1	23.0	7.8	12.3
Thr	ACT	13.1	7.6	9.3	14.8
Thr	ACC	18.9	23.0	6.2	9.8
Val	GTG	28.1	43.4	7.8	12.3
Val	GTA	7.1	7.6	6.2	9.8
Val	GTT	11.0	2.5	3.1	4.9
Val	GTC	14.5	20.4	6.2	9.8
Trp	TGG	13.2	5.1	4.7	7.4
Tyr	TAT	12.2	23.0	4.7	7.4
Tyr	TAC	15.3	20.4	3.1	4.9

[a]Details described under Methods.
[b]*EBNA1* ORF without *GAR*.

 © 2022. The Author(s).
